# The relationship between abdominal fat and sleep quality after combined exercise in patients with type 2 diabetes mellitus

**DOI:** 10.3389/fendo.2025.1471608

**Published:** 2025-06-27

**Authors:** Yu Han, Yue-Xia Han, Fang Huang, Hui-Ming Zou, Qing Gu, Xue Hu, Jing-Xian Fang, Jian Meng, Sui-Jun Wang

**Affiliations:** Yangpu District Shidong Hospital of Shanghai, Endocrinology and Metabolism, Shanghai, China

**Keywords:** type 2 diabetes mellitus, exercise, VAT (visceral adipose tissue), SAT (subcutaneous adipose tissue), sleep quality

## Abstract

**Background:**

Exercise holds promise as a non-pharmacological intervention for improving sleep quality. Therefore, this study investigated the potential relationship between abdominal fat and sleep quality after combined exercise in patients with T2DM (Type 2 diabetes mellitus).

**Material and methods:**

A total of 100 adults with T2DM (52 women, 48 men), aged 55-75years, were enrolled in the study who were sufficiently active and physically fit were recruited and were randomized equally into four groups: a control group, an AEX group (aerobic exercise), a REX group (resistance exercise) and a COMB group (combination exercise). Patient sleep monitoring reports were collected using a smart bracelet. After 12 weeks of intervention, the Pittsburgh Sleep Quality Index (PSQI) total score, total sleep time, sleep efficiency, time to reawakening, BMI (body mass index), FBG (fasting blood glucose), HOMA-IR (insulin resistance index), HbA1c (glycosylated hemoglobin), VAT (visceral adipose tissue), SAT (subcutaneous adipose tissue), and LBM (lean body mass) were compared among the four groups.

**Results:**

All exercise training programs improved the PSQI score in patients with type 2 diabetes. COMB is the only training that improves objective sleep quality and quantity, including total sleep time, sleep efficiency, and wake time after sleep onset.

**Conclusion:**

A comprehensive exercise program to reduce abdominal fat can benefit metabolic and sleep health in people with type 2 diabetes.

## Introduction

T2DM (Type 2 diabetes mellitus) is a long-term metabolic condition primarily defined by high blood glucose levels due to insulin resistance and/or a relative lack of insulin. This condition frequently results in a combination of other health problems, including abdominal obesity and sleep disorders (1, 2).

Abdominal fat accumulation, particularly visceral fat, is strongly linked to insulin resistance and increased cardiovascular risk in patients with T2DM (3). On the other hand, short sleep duration has been linked to various metabolic changes that can promote the development of T2DM (4). Sleep plays an essential role in maintaining overall health and well-being. Sleep disorders can indeed have significant negative impacts on a range of lifestyle-related diseases, including metabolic syndrome, hypertension, diabetes, and cardiovascular disease (5). Studies on the relationship between changes in sleep duration (from short to regular sleep or from normal to long sleep) and the development of T2DM do have inconsistent results (6, 7). A growing body of research suggests that poor sleep quality is strongly associated with disease control and quality of life in T2DM (8).

In recent years, increasing attention has been on the benefits of exercise for patients with T2DM. Exercise is considered an essential component of lifestyle management for T2DM, as it can help improve blood sugar control, reduce body weight, increase insulin sensitivity, and improve cardiovascular health. Combined exercise, specifically aerobic and resistance exercise, is often recommended for T2DM patients (9). Absolutely, there is a strong correlation between physical activity and improved sleep quality (10).

Women and men exhibit typical differential characteristics at the level of body composition and muscle fiber type distribution (11). The body composition of the human body undergoes significant changes with age, primarily characterized by a decrease in lean body mass (muscle mass) and an increase in body fat (12). Men lose muscle mass more rapidly and gain body fat, particularly visceral fat, due to declining androgen levels (13). In women, as estrogen levels fall after menopause, fat distribution shifts from the hips and thighs to the abdomen, and the body fat percentage increases, but muscle loss is relatively minor (14).

It is well known that age and gender factors play a role in the transition of sleep stages in humans. A related study shows that men seem to sleep for shorter periods than women, and that men are less efficient at sleeping than women. Regarding the general population, women have a longer latency period before entering a sleep state (15). In addition, nearly half of postmenopausal women report problems with insomnia (16).

Given these complexities, understanding gender-specific responses to combined exercise is crucial for optimizing the management of type 2 diabetes mellitus (T2DM). Gender differences moderate the body’s adaptive responses to exercise interventions, with factors such as hormone levels, inflammatory responses, fatigue levels, muscle fiber type, and energy metabolism all being involved (17). Given this, gender-specific intervention strategies must be fully considered when developing exercise programs to reduce muscle loss.

However, despite evidence that exercise improves glycemic control and quality of life in patients with T2DM, there is a lack of research on the effect of combined exercise on the relationship between abdominal fat and sleep quality in these patients.

This study investigated the potential relationship between abdominal fat and sleep quality after combined exercise in patients with T2DM. We hypothesized that combined exercise could improve sleep quality in T2DM patients by reducing abdominal fat accumulation. By analyzing this relationship in depth, we expect to provide more scientific and practical guidance for exercise interventions in patients with T2DM, thereby improving their overall health and quality of life. By comparing the changes in data before and after exercise, we will reveal the effect of combined exercise on the relationship between abdominal fat and sleep quality in patients with T2DM and explore its possible mechanisms.

## Methods

### Study design and methods

This study was a randomized, controlled, open clinical trial. All participants signed an informed consent form, and the Institutional Review Boards of all participating institutions approved the study (2024-013-01). The study was registered on the Chinese Clinical Trial Register, ChiCTR2200057863(19/03/2022).

### Study participants

The inclusion criteria of this study were: (1) diagnosis of T2DM. (2) HbA1c 6.5-13.0%. (3) the age range of 55–75 years.

Participants were excluded from the study if they met any of the following conditions: (1) having a physical health condition that precludes regular engagement in physical activity; (2) having severe retinopathy; (3) experiencing cognitive impairment.

The criteria for withdrawal are as follows: (1) loss of visits; (2) occurrence of a serious adverse event; (3) inability to adhere to the intervention medication as required by the trial.

The sample size was calculated based on the results of a prior meta-analysis. Chi, I., et al. (18). Based on calculations using PASS 15.0 software, it was determined that a minimum of 25 participants per group would be necessary, accounting for an anticipated 15% dropout rate. To achieve 80% statistical power and maintain a two-tailed significance level of 0.05, statistical variance analyses were performed with a total sample size of 100 participants.

Participants who were sufficiently active and physically fit were recruited and randomized equally into four groups: a control group, an AEX group (aerobic exercise), a REX group (resistance exercise), and a COMB exercise group (combination exercise).

All participants signed an informed consent, and the Institutional Review Boards of all participating institutions approved the study.

### Intervention methods

The intervention method was conducted as follows.

The control group received regular diabetes treatment, including diet amendments, regular and balanced exercise, and abstinence from smoking and alcohol. According to their condition, metformin tablets or subcutaneous injections of insulin aspartate were delivered to control blood sugar in the required range. For participants with hypertension and hyperlipidemia, this was combined with valsartan, atorvastatin, and other drugs to control blood pressure and blood lipid levels in normal ranges.

The aerobic exercise group performs Tai Chi, while the resistance group performs elastic band exercises. The COMB group performs both Tai Chi and resistance exercise. Each exercise intervention group attended exercise sessions three times per week, lasting 60–90 minutes, with 15 minutes of warm-up and cool-down included.

We standardized the aerobic exercise prescription based on body weights of 41.8 (10) kilojoules (kcal) (COMB) and 50.2 (12) kilojoules (kcal) (AEX) kg body weight–1 week–1 of moderate exercise. In the COMB group, participants were asked to complete resistance training twice per week, consisting of one set of strength-training maneuvers per set and 41.8 (10) kJ (kcal) kg body weight-1 (week-1) burned through aerobic exercise. Elastic band training exercises include chest clamps, deep Squat Push-Ups, and elastic band standing lateral flexion.

We used the Borg Physical Exertion Scale to estimate participant fatigue and maintained the intensity of each exercise at a level of 3-5 (19).

All exercise sessions were supervised by exercise physiologists at our facility. During monthly visits, the control group attended educational sessions about a healthful diet and were asked not to participate in external weight loss or exercise programs.

### Outcomes

General clinical information [gender, age, WC (waist circumference), height, weight, and blood pressure was collected from medical history. Blood measurements included FBG (fasting blood glucose), HbA1c (glycosylated hemoglobin), Fasting C-peptide, TC (total cholesterol), TG (triglycerides), ALT (alanine aminotransferase), AST (aspartate aminotransferase), UA (uric acid), Cr (creatinine), BUN (urea nitrogen), and other biochemicals. BMI (Body mass index) is determined by the formula weight (kg)/height (m)^2^.

Magnetic resonance imaging (1.5T HDxt MRI system) was performed using a standard array coil to measure the patient’s visceral adipose tissue (VAT) and subcutaneous adipose tissue (SAT) in the supine position, localized for imaging at the L4-L5 disc plane, and analyzed using Slice Omatic 5.0. Specifically, the area of visceral fat and subcutaneous fat in the image of the abdominal navel section, in square centimeters (cm²). DXA (Dual-energy X-ray absorptiometry) was performed using a Discovery W Bone Densitometer (Hologic) to assess whole-body lean body mass (LBM).

Sleep quality and quantity were assessed before and after the training program (week 12) using the PSQI scale and a sleep monitoring bracelet (20). Sleep quality was assessed using the Chinese version of the Pittsburgh Sleep Quality Index (C-PSQI, Cronbach’s α = 0.77) (21).

The PSQI is a widely used and well-validated instrument for assessing sleep quality. It is a self-rated questionnaire that evaluates sleep quality and patterns over the past month. Here is a summary of the seven domains you mentioned and how they are assessed in the PSQI:

a. Subjective Sleep Quality: Participants rate their overall sleep quality on a scale ranging from 0 (very good) to 3 (very bad). This domain captures the individual’s perception of their sleep quality.
b. Sleep Latency: Sleep latency refers to the time it takes to fall asleep after going to bed. In the PSQI, participants rate their sleep latency on a scale of 0 (≤15 minutes) to 3 (>60 minutes).
c. Sleep Duration: This domain assesses the sleep obtained each night. Participants rate their sleep duration on a scale of 0 (≥7 hours) to 3 (<5 hours).
d. Sleep Efficiency: Sleep efficiency is calculated as a percentage of time spent asleep in bed. The PSQI asks about the total time spent in bed and the total time spent sleeping, and then calculates the efficiency based on these responses. The scale ranges from 0 (≥85%) to 3 (<65%).
e. Sleep Disturbances: Participants rate the frequency of problems during sleep, such as awakening during the night or early morning, on a scale of 0 (none) to 3 (≥3 times per week).
f. Use of Sleep Medication: This domain assesses the frequency of medication used to help sleep. Participants rate their use of sleep medication on a scale of 0 (never) to 3 (≥3 times per week).
g. Daytime Dysfunction: Daytime dysfunction refers to problems that occur due to poor sleep, such as fatigue or difficulty concentrating. Participants rate the severity of these problems on a scale of 0 (none) to 3 (severe).

The total PSQI score is calculated by summing the scores from all seven domains, ranging from 0 to 21. A higher score indicates worse sleep quality. Participants with C-PSQI scores above 5 in the study were classified as “poor sleep quality” individuals (22).

The PSQI has been extensively used in research and clinical settings to evaluate sleep quality in various populations, including patients with chronic diseases and the general population (23).

Objective characteristics of sleep-wake cycles were monitored with a sleep-monitoring bracelet. Daily, participants utilized a sleep-tracking bracelet (Honor Band 5i by Huawei, based in Shenzhen, China) to capture objective sleep data. This sleep-tracking bracelet is a wrist-mounted intelligent gadget, incorporating cardiopulmonary coupling (CPC) technology that facilitates highly accurate assessments of sleep patterns (24). The bracelet’s accelerometer sensor measures movement in three dimensions (x, y, and z axes), and the device’s software processes this data to infer sleep and wakefulness periods based on the user’s activity level.

A daily professional check of the bracelet ensures it is worn correctly and that the battery is fully charged. This step is crucial for maintaining the accuracy and reliability of the bracelet’s data. Proper wearing and battery charge are essential for accurate monitoring. The bracelet’s ability to automatically collect data for at least 72 hours allows for continuous monitoring of a patient’s sleep-wake cycles over a relatively long period. This eliminates the need for frequent manual intervention and ensures data is captured consistently. The bracelet automatically records the following data: total sleep time (minutes slept between bedtime and wake time), sleep efficiency (percentage of time asleep while in bed), and wake after sleep onset (minutes awake between sleep onset and wake time).

### Statistical analysis

IBM SPSS 26.0 was applied for data processing. Measurement data were described as the mean ± standard deviation. The data was represented by frequency and percentage. Multiple group comparisons were analyzed using one-way ANOVA. Repeated-measures ANOVA was used to determine changes in total PSQI score, total sleep time, sleep efficiency, arousal after sleep onset, and PSQI sub-scores across time and between groups. Time × group interactions were also analyzed. We found a gender interaction in the total sleep time results; Therefore, we repeated the previous analyses by gender. An independent samples t-test was used to test the differences between the groups.

Men and women have significant biological differences in physiology, hormone levels, and metabolic rates. These differences may cause them to respond differently to the same influences. For example, in terms of body composition, men and women typically have different patterns of body fat distribution, with men tending to have more visceral fat, whereas women may differ in subcutaneous fat. Such differences may affect the relationship between body composition and indicators such as sleep quality and glucose metabolism. Considering gender in regression analyses allows for a more accurate assessment of the relationship between these variables and avoids bias caused by ignoring gender differences (25).

The relationship between anthropometric variables, including body composition data, and gender-specific tertiles of sleep quality (as the independent variable) was investigated through linear regression analysis. Differences were considered statistically significant at P<0.05.

## Result

**Figure 1:**
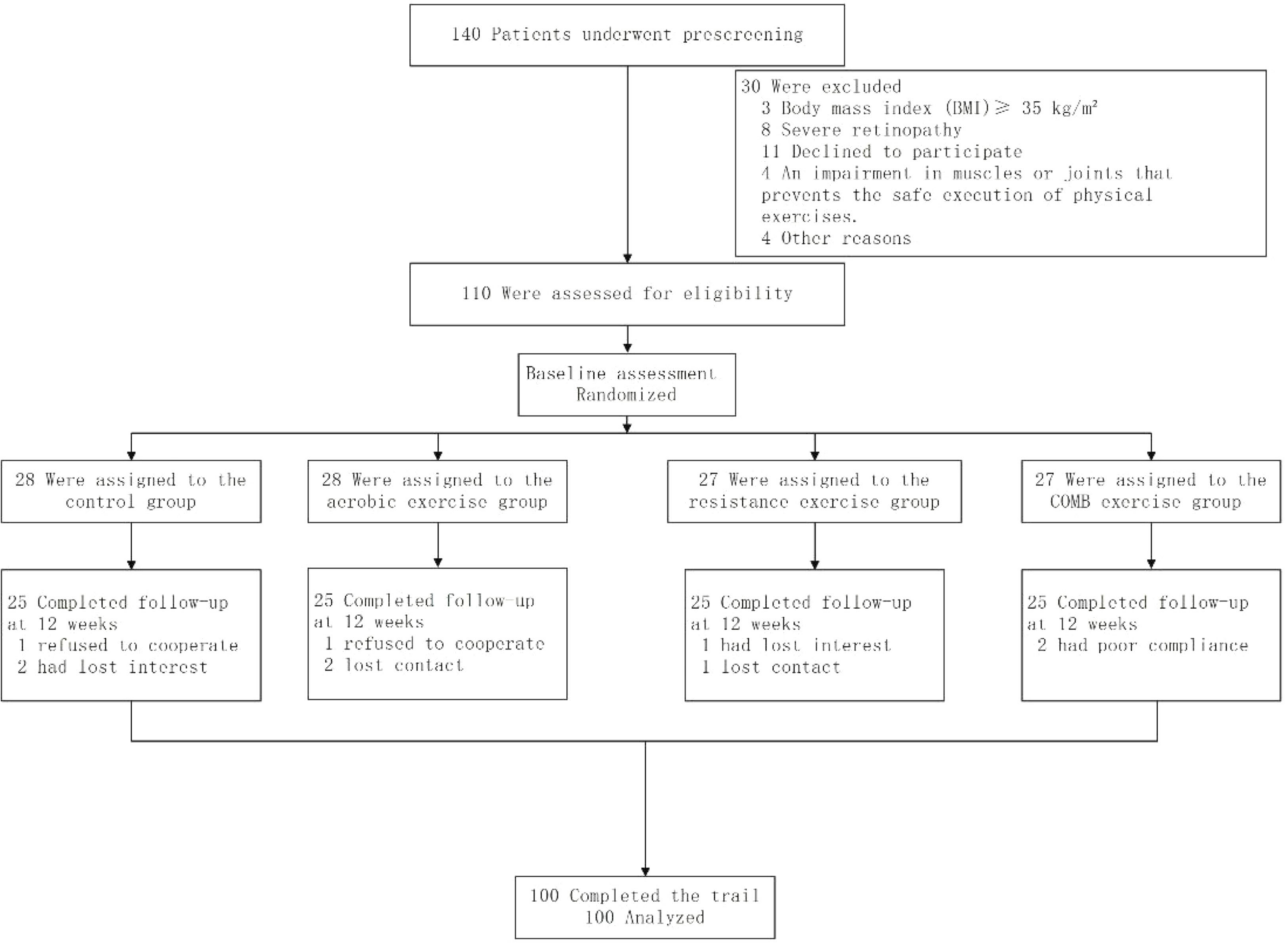
Flow diagram of the study participants.

**Figure 1** shows the participant’s flowchart. 140 participants were screened at baseline, of whom 110 met the inclusion criteria and agreed to participate in the trial. The 110 participants were divided into four groups.

**Table 1** shows the participants’ baseline characteristics. There were no appreciable differences in baseline characteristics among any of the groups.

**Table 1:** Baseline characteristics of the participants.

Characteristic	CON (n = 25)	AEX (n = 25)	REX (n = 25)	COMB (n = 25)	p Value
Age, years	64 ± 7	62 ± 5	61 ± 5	61 ± 6	0.33
Sex, number (%)					
Male	12 (48)	10 (40)	15 (60)	11 (44)	0.64
Female	13 (52)	15 (60)	10 (40)	14 (56)	0.34
Weight (kg)	67.6 ± 6.0	67.4 ± 3.8	68.3 ± 3.9	70.7 ± 6.3	0.72
WC(cm)	93.1 ± 11.6	100.2 ± 14.9	100.0 ± 15.1	103.4 ± 7.6	0.37
BMI (kg/m^2^)	28.7 ± 1.9	28.2 ± 1.2	28.8 ± 2.0	28.7 ± 1.7	0.47
HOMA-IR	4.8 ± 0.9	5.3 ± 0.7	4.3 ± 0.6	4.9 ± 0.7	0.66
SAT (cm^2^)	143.7 ± 14.8	144.9 ± 11.1	143.8 ± 10.6	141.1 ± 7.5	0.15
VAT (cm^2^)	122.8 ± 8.0	134.9 ± 5.8	123.8 ± 6.9	135.1 ± 3.5	0.58
LBM (kg)	33.7 ± 3.8	33.6 ± 4.2	34.2 ± 3.6	34.1 ± 4.1	0.42
Sleep parameters					
PSQI global score	5.8 ± 3.1	6.1 ± 3.2	6.3 ± 2.9	5.8 ± 3.2	0.59
Total sleep time (min)	378.4 ± 53.1	417.5 ± 41.9	399.3 ± 39.2	410.1 ± 44.8	0.27
Sleep efficiency (%)	78.6 ± 6.3	76.1 ± 5.5	84.2 ± 7.2	83.9 ± 5.9	0.52
Wake after sleep onset (min)	72.8 ± 20.4	65.9 ± 19.9	65.2 ± 22.7	71.9 ± 23.7	0.31
HbA1C (%)	8.27 ± 1.22	8.58 ± 0.95	8.14 ± 1.27	8.63 ± 0.97	0.55
FBG (mmol/l)	7.26 ± 1.00	8.08 ± 1.24	7.32 ± 1.13	7.68 ± 0.81	0.68
Total cholesterol (mmol/L)	6.91 ± 1.41	5.98 ± 1.29	6.94 ± 1.36	6.60 ± 1.21	0.31
Triglyceride (mmol/L)	2.88 ± 0.54	2.57 ± 0.84	2.37 ± 0.69	2.64 ± 0.74	0.38
LDL cholesterol (mmol/L)	4.27 ± 0.47	3.67 ± 0.79	3.69 ± 0.88	3.49 ± 0.89	0.18
HDL cholesterol (mmol/L)	1.25 ± 0.19	1.49 ± 0.32	1.38 ± 0.28	1.61 ± 0.35	0.61

Data are presented as mean ± standard deviation.

WC, waist circumference; BMI, Body Mass Index; HOMA-IR, insulin resistance; SAT, subcutaneous adipose tissue; VAT, visceral adipose tissue; LBM= lean body mass; HbA1C, glycated hemoglobin; FBG, fasting blood glucose; LDL, low density lipoprotein; HDL, high density lipoprotein; CON, control; AEX, aerobic exercise group; REX, resistance exercise group; COMB, combined aerobic and resistance exercise group.

Men showed a higher B coefficient in comparison to women (**Table 2**, non-adjusted model). This B coefficient decreased when the model was adjusted for age (**Table 2**, adjusted model).

**Table 2:** Linear regression analysis for the association of percentage of baseline of Pittsburgh Sleep Quality Index (PSQI) and anthropometric and body composition variables and indicators of glucose metabolism before and after COMB exercise.

Variables	Male				Female			
	Unadjusted model		Adjusted model		Unadjusted model		Adjusted model	
	B coefficient(95% CI)	P	B coefficient (95% CI)	P	B coefficient(95% CI)	P	B coefficient(95% CI)	P
BMI	3.67 (-3.49, 5.94)	<0.001	2.73 (0.98, 4.83)	0.032	2.11 (-3.49, 5.94)	<0.001	2.34 (1.28, 11.86)	0.018
WC	2.56 (1.30, 4.71)	0.236	4.70 (-1.51, 6.811)	0.019	1.56 (-2.30, 3.71)	0.411	2.70 (1.51, 4.81)	0.012
SAT	1.72 (0.62, 3.90)	0.372	2.55 (1.41, 3.61)	0.623	1.49 (0.62, 2.78)	0.015	2.03 (1.23, 5.89)	0.011
VAT	4.22 (1.18, 6.45)	0.497	1.15 (1.09, 1.38)	0.853	3.29 (0.66, 7.09)	<0.001	1.93 (0.32, 4.74)	<0.001
LBM	-1.02 (-2.39, 2.85)	0.294	-2.99 (-4.09, 1.76)	0.411	-2.22 (1.18, 6.45)	0.032	-1.51 (-2.69, 2.77)	0.004
FBG	2.54 (1.32-3.02)	0.024	3.37 (1.39, 9.06)	0.018	1.98 (1.06-3.45)	0.019	2.93 (0.41, 6.31)	0.032
HbA1C	3.63 (-1.49, 5.89)	0.017	3.31 (-1.26, 7.99)	0.028	2.61 (-0.85, 6.71)	0.011	3.81 (1.25, 5.51)	0.023
HOMA-IR	3.78 (-5.52, 5.91)	0.667	5.65 (-3.96, 6.95)	0.794	4.18 (0.55, 6.71)	0.681	2.35 (0.91, 6.68)	0.548

Model was adjusted for age.

BMI, Body Mass Index; WC, waist circumference; SAT, subcutaneous adipose tissue; VAT, visceral adipose tissue; LBM= lean body mass; FBG, fasting blood glucose; HbA1C, glycated hemoglobin; HOMA-IR, insulin resistance.

In the unadjusted model, the association between WC and PSQI baseline percentage was insignificant in males (P=0.236) and females (P=0.411). After adjustment, the association became significant in males (P = .019) and females (P = .012) (**Table 2**). Significant associations were found between changes in SAT (P<0.001), VAT changes (P<0.001), and changes in PSQI only in females (**Table 2**).

In the unadjusted model, the association of LBM with PSQI baseline percentage was insignificant in males and females. After adjustment, the association became significant in women (P = .004) (**Table 2**).

In both unadjusted and adjusted models, FBG and HbA1C were significantly and positively associated with PSQI baseline percentage in both men and women (**Table 2**).

PSQI scores decreased in the AEX, REX, and COMB groups compared with the control group (P = .031), with more significant decreases in the REX and COMB groups. Total sleep time increased significantly in the REX group (P = .015), followed by the COMB and AEX groups. Still, the change was not statistically significant in the control group. BMI decreased in the AEX, REX, and COMB groups (P = .017), with more substantial decreases in the REX and COMB groups. SAT (P = .012) and VAT (P = .009) were significantly reduced in both the REX and the COMB groups (**Table 3**).

**Table 3:** Biochemical and imaging parameters in type 2 diabetic patients after intervention with different exercise patterns.

Outcome Variables	CON Difference and 95% CI	AEX Difference and 95% CI	REX Difference and 95% CI	COMB Difference and 95% CI	p Value* Group–Time Interaction	AEX vs CON	REX vs CON	AEX vs REX	COMB vs AEX	COMB vs REX
PSQI global score										
Change at 12 weeks	-0.12(-0.16,0.05)	-0.26(-0.33,0.11)	-0.33(-0.39,-0.01)	-0.37(-0.41,-0.15)	0.031	0.021	0.015	0.341	<.001	<.001
Total sleep time (min)										
Change at 12 weeks	10.0(8.3,15.3)	11.2(5.5,16.9)	32.7(25.7,40.1)	21.5(12.6,26.3)	0.015	0.332	<.001	<.001	0.017	0.025
Sleep efficiency (%)										
Change at 12 weeks	-0.4(-0.9,1.1)	6.9(2.5,8.8)	10.0(4.9,15.3)	11.3(7.7,14.4)	0.038	0.217	0.015	0.362	0.331	0.246
Wake after sleep onset (min)										
Change at 12 weeks	-4.5(-7.2,-0.5)	2.5(-0.3,3.3)	-3.7(-5.1,2.9)	-6.8(-10.9,1.1)	0.024	0.117	0.218	0.294	0.013	0.022
BMI (kg/m2)										
Change at 12 weeks	-0.03(-0.12,0.05)	-2.66(-3.09,0.01)	-2.89(-3.17,-1.03)	-2.72(-3.24,-1.55)	0.017	<.001	<.001	0.335	0.169	0.132
LBM (kg)										
Change at 12 weeks	0.16(-1.21, 0.44)	-0.12(-0.29, 0.22)	8.83(4.33, 10.76)	-0.14(-0.39, 0.34)	0.022	0.316	<.001	<.001	0.339	<.001
SAT (cm2)										
Change at 12 weeks	-2.52(-3.09,1.06)	-11.51(-15.28,-2.88)	-16.97(-20.37,-5.16)	-17.58(-21.93,-6.31)	0.012	0.016	<.001	0.286	0.018	0.361
VAT (cm2)										
Change at 12 weeks	1.52(0.05,1.99)	-22.65(-30.68,-8.03)	-24.21(-32.18,-11.15)	-25.58(-30.43,-10.86)	0.009	0.015	0.022	0.341	0.228	0.371

BMI, Body Mass Index; SAT, subcutaneous adipose tissue; VAT, visceral adipose tissue; LBM= lean body mass; CON, control group; AEX, aerobic exercise group; REX, resistance exercise group; COMB, combined aerobic and resistance exercise group.

**Figure 2:**
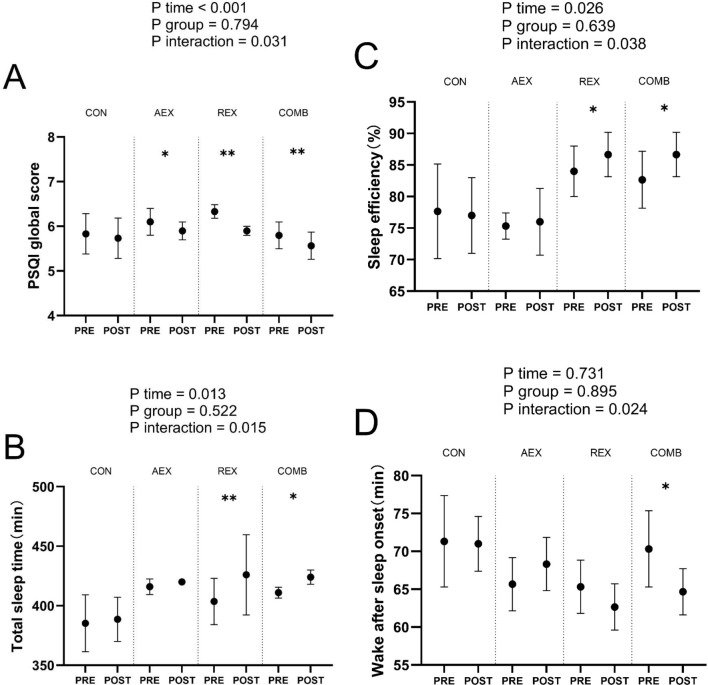
Sleep parameters in type 2 diabetic patients after intervention with different exercise patterns. **(A)** displays reported PSQI global score, **(B)** displays reported total sleep time, **(C)** displays reported sleep efficiency, **(D)** displays reported wake after sleep onset. P values for ANOVA (time, group, and interaction [time × group]) were repeated for all enrolled populations. *P < .05, **P < .01, Student’s paired t test. Data are shown as means ± standard deviation. CON, control; AEX, aerobic exercise group; REX, resistance exercise group; COMB, combined aerobic and resistance exercise group; PSQI, Pittsburgh Sleep Quality Index.

**Figure 2** shows the total PSQI score (**Figure 2A**), total sleep time (**Figure 2B**), sleep efficiency (**Figure 2C**), and sleep quality (**Figure 2D**). Total sleep duration (**Figure 2B**), sleep efficiency (**Figure 2C**), and arousal after sleep onset (**Figure 2D**). Compared to baseline levels, total PSQI scores decreased significantly in all intervention groups, while no differences were observed in the control group. The COMB group showed substantially higher total sleep time, higher sleep efficiency, and lower wake after sleep onset after the intervention program compared to the baseline. Time × group interaction was found in PSQI global score, total sleep time, sleep efficiency, and wake after sleep onset.

**Figure 3:**
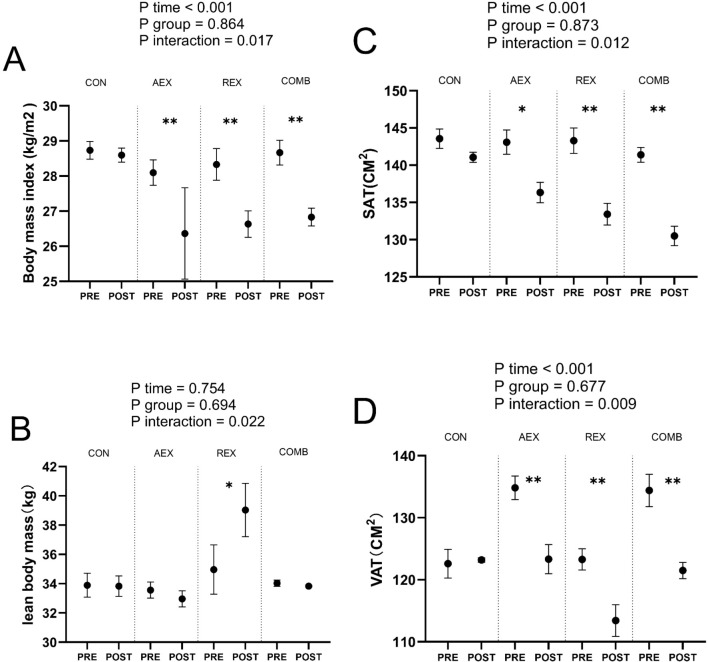
Mean changes in Body Mass Index (BMI) **(A)**, lean body mass (LBM) **(B)**, abdominal subcutaneous adipose tissue (SAT) **(C)**, and visceral adipose tissue (VAT) **(D)** in type 2 diabetic patients after intervention with different exercise patterns. P values for ANOVA (time, group, and interaction [time × group]) were repeated for all enrolled populations. *P < .05, **P < .01, Student’s paired t test. Data are shown as means ± standard deviation. CON, control; AEX, aerobic exercise group; REX, resistance exercise group; COMB, combined aerobic and resistance exercise group.

**Figure 3** shows changes in Body Mass Index (BMI) (**Figure 3A**), lean body mass (LBM) (**Figure 3B**), abdominal subcutaneous adipose tissue (SAT) (**Figure 3C**), and visceral adipose tissue (VAT) (**Figure 3D**).BMI, SAT, and VAT were significantly lower in the COMB group after the intervention program than at baseline.

## Discussion

Based on the data from this study, we can summarize the following points: (1) All exercise training programs improved sleep quality: whether AEX, REX, or COMB, these exercise training programs improved the total PSQI score. This suggests that exercise has a positive effect on improving sleep quality in T2DM. (2) COMB was the only training that improved objective sleep quality and quantity: of all the training groups, only the COMB group improved objective sleep quality and quantity at baseline levels, including total sleep time, sleep efficiency, and wake after sleep onset. (3) Notably, men in the exercise group improved their total sleep time after the intervention, but women did not show the same improvement. This may be due to gender differences playing a role in the effects of exercise on sleep, and further research may be needed to explore this finding. (4) Significant associations were found between changes in body mass index (BMI) and PSQI after combined exercise in both genders and between changes on SAT and VAT and PSQI only in women. (5) Significant associations were found between changes in PSQI and changes in HbA1c and FBG before and after combined exercise.

Sleep duration is a risk factor for T2DM (26). All training groups (AEX, REX, and COMB) improved PSQI global scores by 30.28%, 36.71%, and 40.96%, respectively, improving subjective sleep quality. These findings are consistent with a meta-analysis that reported that different types of exercise, including single-component and combined exercise, were associated with improvements in the Pittsburgh Sleep Quality Index (PSQI). This means that performing a single or combining multiple forms of exercise can help improve sleep quality (27). Regular exercise training has positively impacted overall sleep quality and efficiency (28). Of all the training groups, only the COMB group improved objective sleep quality and quantity, including total sleep time, sleep efficiency, and post-sleep awakening time, at the baseline level. This further supports the unique benefits of COMB training in improving sleep quality (29). The effectiveness of COMB training in improving sleep may be related to its ability to reduce psychological stressors such as stress, anxiety, and depression. COMB training may also indirectly improve sleep quality by enhancing physical fitness. A strong correlation exists between physical fitness and sleep quality, and a strong body tends to support better sleep (30).

It has been established that regular exercise in T2DM can help control blood sugar by increasing muscle glucose uptake and reducing insulin resistance (31). Exercise is widely recognized as having significant benefits for improving sleep quality, but the exact mechanisms behind this are not fully understood. One of the mechanisms by which exercise affects sleep quality is the ability of exercise to regulate melatonin levels (32). Melatonin has direct and indirect physiological regulatory effects on the central nervous system, improving sleep quality, shortening the time to sleep, reducing the number of nocturnal awakenings, and prolonging the deep sleep phase (33). Research shows that exercise regulates melatonin levels, positively affecting sleep quality (34, 35).

Several studies have shown a significant negative correlation between sleep duration, quality, and obesity (36, 37). Several explanations have been proposed for the negative correlation between sleep duration/quality and obesity, with a significant factor being changes in eating behavior due to sleep deprivation (38).

Several observational studies have reported an association between sleep duration and VAT mass. The Korean Genome and Epidemiology Study investigated the effect of different sleep durations on the risk of visceral obesity by analyzing cross-sectional data. The study compared the incidence of visceral obesity among people with varying sleep durations, using visceral fat area ≥100 cm² as the defining criterion for visceral obesity. It was found that the risk of visceral obesity was significantly higher in those who slept less than 5 hours per day than in those who slept more than 7 hours (39). Regarding the study of sleep duration and increased VAT, a long-term observation of 293 participants (for 6 years) provided valuable insights. The study found that participants who slept less than or equal to 6 hours per day at baseline (at the start) had a more significant increase in visceral fat over the 6-year observation period than those who slept 7 to 8 hours per day (40).

Several potential mechanisms could explain the causal relationship between short sleep duration and VAT accumulation.

First, a biopsy study in normal-weight adults suggested that acute sleep deprivation increases levels of proteins associated with fat accumulation in adipose tissue (41). These findings suggest that sleep deprivation promotes anabolic pathways in adipose tissue.

Second, the shift from catabolic to anabolic hormones due to sleep deprivation is a second potential mechanism for the causal relationship between short sleep duration and VAT. For example, blood levels of catabolic signals (e.g., the adipocyte hormone leptin) are reduced in sleep-deprived normal-weight participants. In contrast, blood levels of anabolic hormones (including the glucagon ghrelin) increase after sleep loss (42).

In addition, a third potential mechanism involves changes in the gut microbiota due to sleep deprivation. After two consecutive nights of partial sleep deprivation, normal-weight adults exhibit an increased ratio of Firmicutes to Bacteroidetes (43). This characterization of the gut microbiota has been proposed to play a role in the development of obesity.

The sex differences in sleep quality are attributed mainly to physiological variations between males and females (16). Hormonal changes, particularly during menstrual cycles, menopause, and pregnancy, can significantly affect women’s sleep patterns. For example, estrogen and progesterone levels can fluctuate during the menstrual cycle, which can lead to sleep disturbances such as insomnia, night sweats, and restless sleep. In contrast, men do not experience these hormonal changes (44).

Testosterone in males and estrogen and progesterone in females play important roles in the behavioral regulation of sleep (45). Studies have shown that women’s sleep status is more susceptible to fluctuations in ovarian steroid levels than men’s (46). In addition, testosterone secretion is significantly associated with the human sleep cycle, with a peak around the onset of the rapid eye movement (REM) sleep phase (47).

Furthermore, the differences in exercise physiology between males and females may also affect sleep quality. While exercise has been shown to improve sleep quality in both sexes, the benefits may be more substantial for men than women. Our study also found an improvement in total sleep time after exercise in men but not women. This may be due to the lower gains in muscle mass, cardiorespiratory fitness, and muscle strength in women, which may limit their ability to achieve the same level of physical exertion and, therefore, the same level of improvement in sleep quality (48).

Sex hormones such as estrogen and progesterone regulate energy metabolism and affect fat distribution and insulin sensitivity (49). Women tend to accumulate fat more easily than men, possibly related to gender-related biological factors such as hormone levels. Estrogen plays an essential role in the female body, contributing to fat accumulation in the hips and thighs. Higher testosterone levels in men increase muscle mass and basal metabolic rate (BMR) (50). This hormonal environment enhances protein synthesis and fat oxidation, which may explain why men often experience more pronounced muscle gains and fat loss from resistance training than women (51). Men tend to accumulate fat in the abdominal region, especially VAT, which is associated with an increased risk of metabolic diseases (52). In our findings on women, a relationship between changes in PSQI, changes in SAT, and changes in VAT only in women. This may be related to the characteristics of female body fat distribution.

Notably, although most previous findings suggest an association between sleep duration and VAT, some studies have failed to confirm these results (53).

The benefits of REX alone in slowing lean body mass loss are also worth noting. This exercise mode may be a suitable option for those unable to complete an aerobic or comprehensive exercise program. However, this study did not find a correlation between LBM and sleep quality.

A meta-analysis of recent cross-sectional studies explored the relationship between sleep duration and HbA1c in patients with T2DM. According to the results of this meta-analysis, there was a significant association between sleep duration and HbA1c levels. In studies exploring the relationship between patients with T2DM and sleep duration, a notable finding was that T2DM patients with habitually short sleep (usually defined as less than 7 hours per night) tended to have higher levels of HbA1c, compared to patients with normal sleep duration (usually 7–8 hours per night) who demonstrated lower levels of HbA1c. This finding reveals that sleep deprivation may negatively affect glycemic control in diabetic patients (54). A study of 16 participants delved into the real-life effects of extended sleep on glucose metabolism, showing a significant positive correlation between changes in sleep duration and fasting blood glucose levels (r = 0.53, P = 0.041). The study also found a significant negative correlation between changes in sleep duration and insulin levels (r = -0.60, P = 0.025) ^6^. Our study also found the positive effect of sleep on blood sugar before and after combined exercise.

Prolonged hyperglycemia-induced neuropathy can lead to autonomic dysfunction, and insulin resistance and blood glucose fluctuations can trigger abnormal secretion of stress hormones such as cortisol and adrenaline, all of which interfere with the sleep-wake cycle (26).

Hyperinsulinemia promotes adipocyte differentiation and inhibits lipolysis, leading to visceral fat accumulation (55). This pattern of fat distribution not only exacerbates insulin resistance but also triggers a systemic inflammatory response through the release of free fatty acids, creating a vicious cycle of obesity and diabetes (56).

Excessive activation of the hypothalamic-pituitary-adrenal axis promotes appetite and inhibits energy expenditure, while abnormal secretion of adipokines such as leptin and adiponectin weakens the body’s satiety signals, leading to excess calorie intake, which also exacerbates the accumulation of abdominal fat (38).

Exercise promotes glucose uptake by increasing the expression of glucose transporter protein 4 (GLUT4) on muscle cell membranes, accelerates muscle utilization of glucose, reduces hepatic glycogenolysis and gluconeogenesis, and attenuates oxidative stress and inflammatory response, delays pancreatic β-cell apoptosis, promotes insulin secretion, reduces insulin resistance, and lowers blood glucose levels (57).

Obesity is a common risk factor for obstructive sleep apnea (OSA) and T2DM (58). Still, an obesity-independent link exists between the two (59). OSA may exacerbate T2DM and lead to hyperglycemia through various mechanisms, with surges in catecholamines and other counter-regulatory hormones being one of the key factors (60).Fat accumulation in the neck of obese people leads to narrowing of the airway, muscle relaxation during sleep, and airway collapse, triggering obstructive sleep apnea (61). When sleep deprivation occurs, leptin secretion decreases and hunger hormone (Ghrelin) secretion increases, causing metabolic disorders (62).

Visceral adipose tissue is a significant risk factor for T2DM and cardiovascular disease (63). Reducing abdominal fat through exercise may improve metabolic health and inflammatory markers (64). Poor sleep in T2DM is linked to hyperglycemia, increased appetite, and impaired glucose tolerance (65). Exercise has been shown to enhance sleep duration and efficiency and reduce fragmentation (66). The current study explored the effects on diabetic sleep and abdominal fat metabolism through different exercise modalities.

### Limitations

The current study suffers from an insufficient sample size and narrow geographic coverage, which makes it difficult to make scientific and comprehensive judgments about differences in cohort characteristics (i.e., cohort effects). To enhance the generalizability and reliability of the study findings, increasing the total sample size and expanding the recruitment scope to multiple geographic regions is recommended. This would effectively weaken the interference of single geographic factors on the study results and reduce the study’s potential geographic bias and limitations.

Second, participants’ diets, stress levels, and other lifestyle habits can confound the relationship between exercise, abdominal fat, and sleep quality.

Third, while exercise is known to reduce abdominal fat and improve sleep quality, the underlying mechanisms (e.g., hormonal changes, inflammation reduction) are not fully elucidated. This limits the ability to tailor interventions effectively.

As a next step, we will establish a more complete remote management system and a mature team to help more patients.

## Conclusion

In conclusion, combined exercise programs targeting abdominal fat reduction may benefit metabolic and sleep health in patients with T2DM. Future research should explore the mechanisms underlying this relationship and optimize exercise programs to maximize these benefits. Additionally, the long-term effects of combined exercise on abdominal fat reduction and sleep quality should be investigated. However, further studies are needed to confirm the observed results in individuals with similar and different characteristics, since the sample size was relatively small.

## Data Availability

The original contributions presented in the study are included in the article/supplementary material. Further inquiries can be directed to the corresponding authors.
